# Tattling Doctors—The Truth in a Nutshell—Consultations

**Published:** 1883-07

**Authors:** 


### HOMŒOPATHIC PHYSICIAN.

A MONTHLY JOURNAL OF MEDICAL SCIENCE.

"If our school ever gives up the strict inductive method of Hahnemann, we are lost, and deserve only to be mentioned as a caricature, in the history of medicine."—CONSTANTINE HERING.

Vol. III. JULY, 1883. No. 7.

### EDITORIAL.

TATTLING DOCTORS.—There can be scarcely a greater indiscretion in a physician than the habit of reporting, from patient to patient, the ailments of those under his care. It often requires much tact to avoid this tattling, and at the same time to avoid giving offense to inquiring *friends*. A physician is often asked: "You are attending Mrs. ———. What is the matter with her?" Such a question, coming from a friend of the family, is hard to dodge, and yet dodged it should always be. No physician has any right to talk of the ailments of his patients. Many do it, and to their own *loss*. Many a time do we hear persons say: "Oh! yes, Dr. ——— is a good physician, but *he talks too much*." These remarks may seem very trite, being upon a subject so universally acknowledged; but we have reason to believe that some of our physicians are too gossipy, too fond of telling of their cases. Some do this from the mere love of gossip; others from a fear of offending their questioners if they decline to answer. A patient may be sure of this: That a physician who will tell them of the ailments of others, will be as likely to tell theirs to some one else. How do they like that prospect? The following, from the late Prof. Chas. D. Meigs, is pertinent to this subject: "Let the student, then, take a firm resolution to guard with good faith those secrets with which he may become acquainted as physician. He should beforehand consider the meaning of the term professional secrets, and know that they are either accidental revelations, or homage due to his station as physician, and not to himself as a person; for of the vast number of those which may be communicated to him or discovered by him, not a tithe or hundredth part of them would be his but for his professional position. If a man be dishonored who reveals a secret communicated by a friend, how far more base is he who takes advantage of his professional standing to make public circumstances that have been intrusted, so to speak, not to himself alone, but to the sacred character of the Iatrist. He disgraces his calling in disgracing himself. It is not in regard to grave and serious matters only that he is called upon to be silent, prudently abstaining from acquiring for himself and his brethren the unenviable character of the babbler; for even the most inconsiderable circumstances as to the sick are confidences that ought not to be *betrayed.*"

---

THE TRUTH IN A NUTSHELL.—In a recent number of a journal a writer, in stating his belief in the efficacy of high potencies, says: "I noticed that the latter (*i. e.*, the low-potency advocates) pay special attention to some other branch of medical science, such as surgery, pathology, or histology. Proficiency in their respective specialties requires so much time that they are forced to neglect therapeutics. I became, therefore, fully convinced of the fact that, if the high potency has not been found universally successful, the fault lies not in the potency, but in the prescriber."

The writer here gives us the truth in a nutshell—that failures with high potencies are due to the *prescriber* every time!

This gentleman, Dr. G. C. Quezada, of Brooklyn, studied homœopathic therapeutics in the only way they can ever be successfully studied. First, by studying Hahnemann's *Organon;* secondly, by diligently studying his *Materia Medica Pura;* thirdly, by making his own potencies and carefully prescribing them. The lower potencies were first tried, then the higher—the true method of testing their relative merits.

---

CONSULTATIONS.—Dr. A. L. Gihon, U. S. N., in a paper read before the American Medical Association at its recent meeting, says a homœopath in seeking a consultation with a divine regular acknowledges his lack of success. Certainly! Why else seek a consultation? In olden time, when homœopaths practiced Homœopathy, they were successful and needed no allopathic assistance. But in these latter days, when "scientific," "liberal" Homœopathy is practiced, failures follow, and so the necessity for old-school assistance. The once great American Institute, now weak and puny through its eclecticism, last year *begged** for such aid, only one physician feeling no need of aid and comfort from the enemy.

* We feel justified in saying that this now famous resolution refers "solely to the consultation question"—*for its author says it does* and he ought to know. If this be its meaning, it is couched in such beautifully uncertain language that even a Talleyrand might envy its ambiguousness.

